# Comparative Examination of Antioxidant Capacity and Fingerprinting of Unfractionated Extracts from Different Plant Parts of Quinoa (*Chenopodium quinoa*) Grown under Greenhouse Conditions

**DOI:** 10.3390/antiox8080238

**Published:** 2019-07-24

**Authors:** Dayana Buitrago, Ivon Buitrago-Villanueva, Ricardo Barbosa-Cornelio, Ericsson Coy-Barrera

**Affiliations:** Bioorganic Chemistry Laboratory, Universidad Militar Nueva Granada, Cajicá 250247, Colombia

**Keywords:** *Chenopodium quinoa*, antioxidants, phenols, flavonoids, fingerprinting, functional foods

## Abstract

Integrated surveys of metabolic profiles and antioxidant capacity from *Chenopodium quinoa* have been limited and have particularly focused on an examination of seeds and leaves. According to this, the main aim of the present study was to address an evaluation of the antioxidant activity of crude ethanolic extracts from different plant parts (leaves, stems, roots, flowers, and seeds) harvested at different times during growth and processed by two distinct drying methods: Air-drying and freeze-drying. In order to characterize the resulting extracts, the total content of phenolics (TPC) and flavonoids (TFC) was then measured through the Folin–Ciocalteu method, while antioxidant capacity was determined using 2,2-diphenyl-1-picrylhydrazyl (DPPH^•^) free radical scavenging and ferric-reducing antioxidant power (FRAP) methods. Parallel to this evaluation, extracts were profiled by LC-DAD-ESI-MS. Data analysis was supported by statistics. Most of the extracts obtained from freeze-dried samples showed higher TPC values ranging from 6.02 to 43.47 milligram of gallic acid equivalents per gram of plant material and a TFC between 1.30 and 12.26 milligram of quercetin equivalents per gram of plant material. After statistical analysis, a low correlation between TPC and TFC values was observed regarding antioxidant capacity from DPPH and FRAP measurements of both drying methods. A multivariate analysis showed that antioxidant components and antioxidant capacity in *C. quinoa* changed during growth and between plant parts and drying methods. These changes need to be taken into consideration when comparing the production/accumulation of beneficial bioactive compounds in this pseudocereal.

## 1. Introduction

Quinoa (*Chenopodium quinoa*) is characterized by the presence of a wide group of phenolic-related phytochemical compounds with antioxidant capacity [1,2], so this can be hence considered as a functional food due to its beneficial effect on the health and wellness of the consumer, beyond its nutritional contribution [3,4,5,6]. The antioxidant function of phenol-containing compounds is due to the presence of a hydroxyl group linked to an aromatic ring, which can provide a defensive barrier against oxidative stress through its inhibitory capacity of reactive oxygen species (ROS) [7,8,9,10,11]. Oxidative stress is present in some pathological stages where cellular functionality is affected, so the evolution of degenerative diseases is promoted [12], such as atherosclerosis and cardiomyopathies, Alzheimer’s and Parkinson’s neurodegeneration [13,14,15], cardiovascular diseases, and cancer [13].

ROS incorporate a wide set of radical and nonradical molecules that act as oxidizing agents or that can be converted into free radicals (FRs) easily [7,9]. FRs and ROS perform an important role in the homeostatic equilibrium, which refers to the normal function of regulatory mechanisms that keep normal physiological conditions within organisms. Among ROS, some examples of FRs can be found, such as superoxide anions (O_2_^−.^), hydroxyls (OH^.^), peroxides (ROO^.^), and nitrogen oxides (NO^.^, NO_2_) [9,16]. The excessive production of ROS or partially reduced compounds, provided with a high chemical reactivity in the cellular structures, can deplete antioxidant defenses, causing several damage that affects biomolecules such as nucleic acids, proteins, carbohydrates, and lipids [13]. The FRs are naturally generated during cellular metabolism in redox reactions, which are carried out mainly inside of the mitochondria and involucrate enzymes such as nicotinamide adenine dinucleotide phosphate (NADPH) oxidase, lipoxygenases, cyclooxygenases, and peroxidases [17,18,19]. The radicals can also originate from external factors, such as the frequent consumption of high-fat-containing and processed foods, excessive alcohol intake, and exposure to diverse chemical agents such as pigments, tobacco smoke, pesticide, and fertilizers, as well as to physical agents such as ionizing radiation employed in radiotherapy, X-rays, UV light, and elevated temperatures [9,20].

Several epidemiological reports and related meta-analyses have recognized a positive correlation between the intake of some vegetables/fruits and health benefits. Such benefits involve the protection/prevention of some of the above-mentioned oxidative-related diseases and even aging [20]. In other words, such foods have been catalogued as functional foods due to their capacity to promote good health through the presence of antioxidants [21]. However, in spite of the plethora of studies on the health benefits of natural antioxidants and their sources, the required daily intake (RDI) must be taken into consideration when consuming them (according to regulations) [22]. Phenolics-related compounds and their sources have a recognized good impact on health as dietary antioxidants [23].

*C. quinoa* seeds exhibit increased nutritional facts due to their elevated contents of vitamins, oils, antioxidants, proteins and amino acids [24,25,26,27], mineral nutrients (e.g., calcium, potassium, phosphorus, magnesium, iron, copper, and zinc) [28,29], and essential fatty acids (e.g., oleic and linoleic acid) and their low content of saturated fatty acids (e.g., palmitic acid) [29,30]. The existing proteins in quinoa exhibit an elevated proportion of some particular amino acids (lysine, histidine, and methionine) in comparison to other cereals, and therefore its consumption gives an adequate nutritional input in a vegetarian diet [31]. Quinoa flour also contains isoflavones such as daidzein and genistein, which have estrogenic or antiestrogenic effects [32]. In addition, it is gluten-free [33,34], making it an alternative food for people who suffer from celiac disease and cereal-related allergies [35,36]. Finally, this plant is also recognized for its antioxidant activity due to the presence of flavonoids and tocopherols, as well as vitamin contents such as thiamin (B1), riboflavin (B2), niacin (B3), and folic acid (B9) [37]. Among the excellent properties and benefits of *C. quinoa* seeds (constituting the main commercial product), quinoa also contains flavonoids that confer antioxidant abilities [26,37]. Flavonoids (e.g., quercetin and kaempferol glycosides) and phenolics (e.g., vanillic and ferulic acids and their derivatives) are the main compounds from quinoa seeds that are directly related to the antioxidant capacity of this plant [38,39]. However, both seeds and pericarp (in a higher proportion) also accumulate some triterpenoids, such as saponins, which act as a plant defense mechanism against insects and parasites. Saponins give a bitter taste, are toxic due to their hemolytic activity, and have antibiotic properties [40,41].

As part of our research on natural antioxidants, the main aim of the present study consisted of the measurement of the antioxidant capacity and the total content of phenolics (TPC) and flavonoids (TFC)) of unfractionated ethanolic extracts obtained from different plant parts of *C. quinoa*. This plant was then cultured under greenhouse conditions, and plant materials were collected at three different times during growth and processed by two distinct drying methods. A subsequent analysis to integrate all of the data was also performed, employing metabolic fingerprinting.

## 2. Materials and Methods

### 2.1. Plant Material and Raw Plant Material Preparation

Twenty-day *C. quinoa* seedlings were placed in flowerpots using a mix of soil and rice husk as substrate (7:3 ratio) under greenhouse conditions on the Bogotá plateau. Average temperature was 18.6 ± 6.1 °C, and relative humidity (RH) was 77 ± 5%. The experimental design was randomly organized in order to harvest the plant material at 1, 3, and 6 months after transplantation. As plants reached harvesting time, plant material was separated into different plant parts: Leaves (Lv), roots (Rt), stems (St), seeds(S), and flowers (Fl). Each plant part was subdivided into two portions, and each portion was processed by a different drying method: (1) The sample was air-dried at 40 °C over 24 h and then crushed, and (2) the sample was first pulverized using liquid nitrogen and subsequently freeze-dried by lyophilization over 8 h. The samples were dried using these methods until they reached constant weight. Dried materials were stored at −20 °C until extraction.

### 2.2. Extract Preparation

Dry plant materials (roots, leaves, stems, flowers, and seeds) were processed three times through ultrasound-assisted extraction using 96% ethanol as a solvent. The resulting mixtures were collected, filtered, and concentrated by reduced pressure using a rotary evaporator. Additionally, the superficial granules from the leaves were also extracted. Thus, these granules were separated from the leaves and extracted with chloroform for 1 h by cold-soaking on a shaker. The extract was then recovered in a vial, and the solvent was removed by reduced pressure at 40 °C. Finally, the crude extracts were weighed and stored at 4 °C until use.

### 2.3. Determination of Antioxidant Capacity, TPC, and TFC of Ethanolic Extracts

DPPH (2,2-diphenyl-1-picryhydrazyl), Folin–Ciocalteu reagent, sodium carbonate, ethyl acetate, formic acid, and absolute ethanol were purchased from Merck (Germany). Trolox, gallic acid, and quercetin were acquired from Sigma (USA). TPC, TFC, and antioxidant capacity assays (both ferric-reducing antioxidant power (FRAP) and DPPH) were carried out on the basis of colorimetric methods [42] scaled down on 96-well microplates. Absorbance measurements were performed on a microplate reader, the Elisa EZ READ 2000, using Galapagos software.

#### 2.3.1. DPPH Radical Scavenging and Ferric Reducing Antioxidant Power (FRAP) Assays

For radical scavenging, ethanolic solutions with variable concentrations (0–500 ppm) of crude ethanolic extracts were prepared: 10 mM of DPPH solution (190 µL) was separately added to each ethanolic extract solution (10 µL). This mixture was incubated at room temperature for 60 min under darkness, and the absorbance at 515 nm was then measured. On the other hand, FRAP reagent solution (190 µL), prepared from a 1:1:10 mixture of solutions of 20 mM FeCl_3_, 10 mM 2,4,6-Tri(2-pyridyl)-s-triazine, and 0.3 M acetate buffer at pH 3.6, were separately added to each ethanolic extract solution (10 µL) with variable concentrations. This mixture was incubated at 37 °C for 30 min, and the absorbance at 593 nm was then measured. Three replicates were evaluated for each determination. Trolox and gallic acid (0–15 ppm) calibration curves (absorbance vs concentration) were also constructed for both the DPPH and FRAP assays to be used as external standards for antioxidant capacity comparison.

#### 2.3.2. Total Phenol Content (TPC) and Total Flavonoid Content (TFC)

For TPC, a set of ethanolic extracts with initial variable concentrations (0–500 ppm) adjusted to an absorbance at 765 nm fell in the range of 0.08–0.8 absorbance units. To 20 µL of adjusted solution were added 10% Folin-Ciocalteu (FC) reagent (40 µL) and 7.35% sodium carbonate (150 µL) solutions. The mixture was incubated under darkness at room temperature for 2 h, and the absorbance of the solutions at 765 nm was then measured. In the case of TFC, an aliquot of ethanolic extract solution (70 µL) was added to a mixture comprised of ethanol (50 µL), 10% aluminum trichloride (10 µL), and 0.1 M sodium acetate (10 µL). Subsequently, the mixture was allowed to react over 40 min under darkness, and finally its absorbance at 420 nm was measured. Three replicates were evaluated for each determination. For TPC and TFC, a quantitation through a standard curve of gallic acid and quercetin was employed, respectively.

#### 2.3.3. Data Analysis

All samples were analyzed in triplicate, and the quantitative results were expressed as mean ± standard deviation. The results were analyzed by ANOVA and the Tukey test at a 95% confidence level within a randomization-based design. The correlation between the different assays was evaluated by a multiple correlations test using Pearson coefficients. All of these statistical analyses were performed using the R 3.6.1 (R Development Core Team, 2019) software package.

### 2.4. Metabolite Fingerprinting

LC/MS-based metabolic fingerprinting was employed for discriminating samples according to recognizable chemical patterns. Thus, chromatographic analyses of ethanolic extracts were performed using a Shimadzu Prominence liquid chromatograph (Shimadzu Corp., Columbia, MA, USA) coupled to an SPD-M20A diode array detector (DAD) and an LCMS-2020 mass spectrometry detector (MSD) equipped with an electrospray (ESI) interface and quadrupole analyzer. A premier C18 column (150 × 4.6 mm, 3.5 μm) was employed for the analysis.

#### 2.4.1. Liquid Chromatography hyphenated with Photodiode-Array Detection and Electrospray Ionization Mass Spectrometry (LC-DAD-ESI-MS) Analysis

Samples were dissolved in absolute ethanol at 2.5 mg/mL concentration and analyzed using the following conditions: A binary mobile phase consisting of 0.005% formic acid in water (solvent A) and acetonitrile (ACN, solvent B) in gradient mode, with 0% of B from 0 to 2 min. Then B was gradually raised to 30% at 11 min and kept at this value for 3 min, and there was subsequently another gradient until 100% of B at 22 min was applied. This composition was held for 3 min, and finally the concentration of B was decreased to 0% at 27 min and kept at this composition until 30 min. The flow rate was 0.5 mL/min, and the injection volume was 20 μL. The monitoring wavelengths were selected at 270 and 330 nm. Mass spectra were simultaneously acquired using electrospray ionization in the positive and negative ion modes (scan 100–2000 *m/z*). A voltage detector of 1.5 kV was used. A curved desolvation line (CDL) and heat block temperature of 250 °C was used. Nebulization gas flow was set at 1.5 L/min. Peak annotations were performed after scrutiny of the mass spectra and UV-vis spectra data of each chromatographic signal in comparison to the information previously reported in literature restricted to the *Chenopodium* genus [39,43].

#### 2.4.2. Multivariate Statistical Analysis

Chromatographic profiles were previously refined with a baseline correction performed with OPENCHROME, were normalized and scaled with EXCEL2013, and were aligned with MATLAB R2013a. These data were then processed with SIMCA 13.0.3 (Umetrics Inc., Umeå, Sweden), which performed an unsupervised principal component analysis (PCA) and a supervised orthogonal partial least squares discriminant analysis (OPLS-DA) and loadings line plots for the searching of chemical patterns related to sample classification.

## 3. Results and Discussion

In this study, the antioxidant capacity and total phenolic and flavonoid content (TPC and TFC) were integrally assessed in the different plant parts (leaves (Lf), stems (St), roots (Rt), seeds (S), and flowers (Fl)) at different harvesting times from transplantation, i.e., 1, 3, and 6 months. Two drying processing methods were also applied to the harvested plant samples, air-drying and freeze-drying. Finally, a metabolic fingerprinting involving all plant parts after 6 months from transplantation was finally carried out from the LC/MS data.

### 3.1. Determination of Total Phenolic Content (TPC)

**Figure 1:**
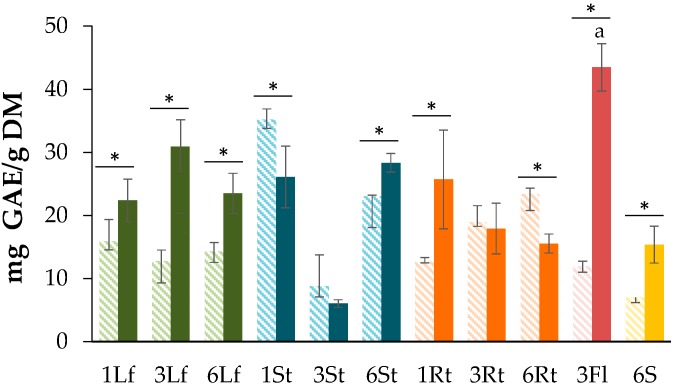
Total phenolic content (TPC) of *Chenopodium quinoa* processed by air-drying (dash bars) and freeze-drying (dark bars) at different times after seeding (1, 3, 6 months) of different plant parts (leaves (Lf), stems (St), roots (Rt), seeds (S), and flowers (Fl)). Each bar represents the mean ± standard deviation (*n* = 3). Significant differences between plant parts (*p* = 0.00432 < 0.01) were found. Letters represent significant differences between treatments analyzed by the Tukey test (*p* < 0.05). * *p* < 0.05 when compared between drying methods.

TPC was measured by means of the Folin–Ciocalteu method and expressed as mg of gallic acid per gram of dry material (mg GAE/g DM). The average values of TPC in the studied plant extracts are shown in Figure 1.

A high content of phenolics was observed in freeze-dried flowers (43.0 ± 3.74 mg GAE/g DM) and seeds (15.4 ± 2.92 mg GAE/g DM) in comparison to roots and stems. Broadly, freeze-dried samples showed more phenolic content than the air-dried ones. In general, there was no clear tendency toward TPC behavior among the plant parts. This could be attributable to the changes in the weather during the culture time, which was characterized by the alternating dry and rainy seasons. Some studies have shown that the total content of phenols and flavonoids is reduced by temperature increases [44,45,46,47,48]. In seeds, the TPC in freeze-dried samples was greater than the air-dried ones, involving 15.0 ± 2.92 and 7.0 ± 0.82 mg GAE/ g DM, respectively: The reduction in TPC during the drying process could be caused by the binding of these compounds to proteins, hindering extraction and/or determination by colorimetric methods [49,50]. In the present work, the measured phenols in seeds obtained under greenhouse conditions were remarkably higher than other values reported previously, such as 0.142 to 0.655 mg of GAE per gram of dry matter [48], 0.352 to 1.399 mg of GAE per gram [51], and 1.11 mg of GAE per gram [52]. The principal feature of this assay was the high content of phenolic compounds in freeze-dried samples compared to the air-dried samples, indicating the use of freeze-drying as a suitable technique for enhanced phenol recovery in extracting methods applied to *C. quinoa* samples. Finally, a particular behavior during growth was observed for TPC in stems, since the extract derived from this plant part, collected the third month after transplantation, showed a lower TPC value compared to stem-derived extracts at 1 and 6 months. This fact can be supported as a consequence of phenolic downregulation due to the elongation of the *C. quinoa* plant at that time (i.e., third month) [53].

### 3.2. Determination of Total Flavonoid Content (TFC)

**Figure 2:**
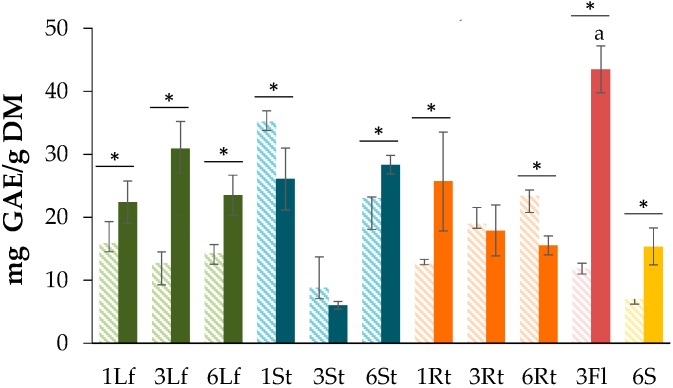
Total flavonoid content (TFC) of *C. quinoa* processed by air-drying (dash bars) and freeze-drying (dark bars) at different times after seeding (1, 3, 6 months) of different plant parts (leaves (Lf), stems (St), roots (Rt), seeds (S), and flowers (Fl)). Each bar represents the mean ± standard deviation (*n* = 3). Significant differences were found between plant parts (*p* = 0.00507 < 0.01), but no differences existed for harvesting times and processing method (*p* = 0.85371 > 0.05). * *p* < 0.05 when compared between drying methods.

The TFC values are expressed as milligram of equivalent quercetin per gram of dry material (mg QE/g DM), and the results show that the extract performed a similar behavior for TFC with respect to TPC, where freeze-dried flowers (12.0 mg GAE/g DM) showed a higher value than other samples (Figure 2).

In seeds, the average TFC values in the freeze-dried samples yielded 5.04 ± 0.20 mg QE/g DM, while in the air-dried seeds the TFC achieved 1.54 ± 0.32 mg QE/g DM, which was about three-fold lower. Nevertheless, the TFC in quinoa seeds could vary depending on the variety examined [54]. Similarly to TPC, stems exhibited the lowest TFC value on extracts from plant material harvested at the third month, which can be rationalized due to plant elongation and/or by a temperature increase in the dry season during that harvesting time.

### 3.3. Evaluation of the Antioxidant Capacity

#### 3.3.1. DPPH Free Radical Scavenging Assay

**Figure 3:**
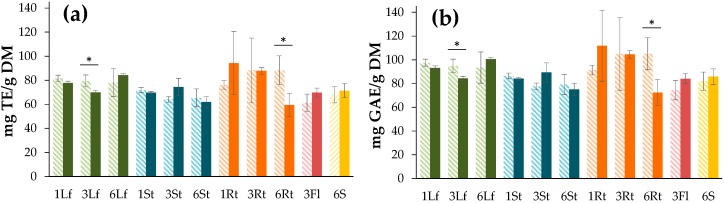
DPPH^•^ free radical scavenging assay of *C. quinoa* processed by air-drying (dash bars) and freeze-drying (dark bars) at different times after seeding (1, 3, 6 months) of different plant parts (leaves (Lf), stems (St), roots (Rt), seeds (S), and flowers (Fl)). Each bar represents the mean ± standard deviation (*n* = 3). No significant differences were found between treatments (*p* = 0.25 > 0.05). (**a**) Trolox as standard, (**b**) gallic acid as standard. * *p* < 0.05 when compared between drying methods.

The antioxidant capacity was evaluated in terms of radical scavenging against a 2,2-diphenyl-1-picrylhydrazyl radical (DPPH assay) and expressed as Trolox equivalents (Figure 3a) or gallic acid equivalents (Figure 3b). The antioxidant capacity, expressed as both Trolox and gallic acid equivalents, exhibited slight differences (ca. 15 units) according to the standard sensitivity, with gallic acid being more sensitive. Thus, the DPPH^•^ radical scavenging capacity was found to range from 72.1 ± 11.1 to 109.5 ± 29.8 mg GAE/g DE, but no significant differences (*p* > 0.05) were observed during growth between plant parts and the drying method, so this capacity was particularly retained along such factors (Figure 3). The impact of the air-drying temperature on *C. quinoa* seeds was studied in a previous work, which found a decrease in antioxidant activity above 50 °C [48]. This fact can rationalize the present outcome related to the similar results using the freeze- and air-drying methods.

#### 3.3.2. Ferric-Reducing Antioxidant Power (FRAP) Assay

**Figure 4:**
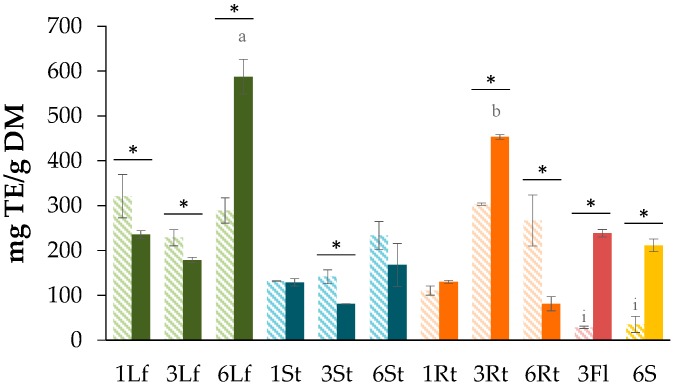
Ferric-reducing antioxidant power (FRAP) assay *C. quinoa* processed by air-drying (dash bars) and freeze-drying (dark bars) at different times after seeding (1, 3, 6 months) of different plant parts (leaves (Lf), stems (St), roots (Rt), seeds (S), and flowers (Fl)). Each bar represents the mean ± standard deviation (*n* = 3). Significant differences between plant parts and harvesting times (*p* = 0.026865 > 0.01). Letters represent significant differences between treatments analyzed by the Tukey test (*p* = 0.05). * *p* < 0.05 when compared between drying methods.

On the other hand, the results for antioxidant capacity in seeds assessed by the FRAP method showed an average of 35.2 ± 17.96 mg trolox equivalent (TE)/g DM for the air-dried samples. The FRAP values for the freeze-dried samples were found to be 211.4 ± 14. 19 mg TE/g DM (Figure 4). These values were noticeably higher than those reported by Repo and Encina (2008) for seeds from *C. quinoa* (values ranging from 0.117 to 2.4 mg TE/g, including different quinoa varieties) [55]. In other reports employing different methodologies, the results showed average values ranging from 1.11–42.3 mg GAE/g in the DPPH and FRAP assays [3,56].

#### 3.3.3. Correlation of TPC, TFC, DPPH, and FRAP Tests

**Figure 5:**
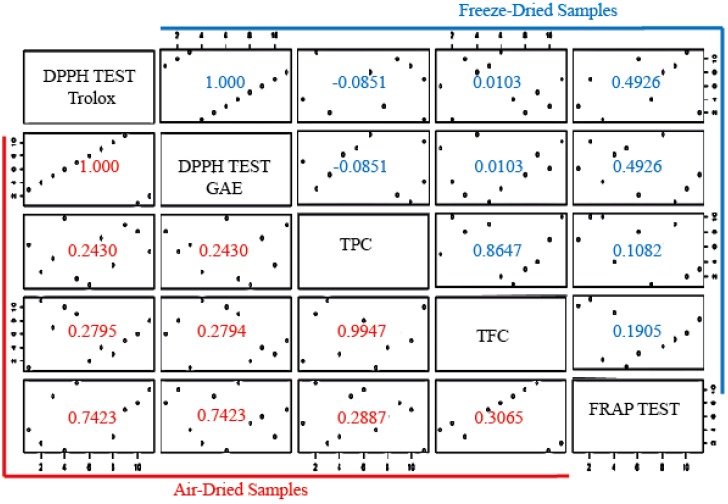
Scatter diagram with Pearson correlation coefficients for freeze- and air-dried samples’ performance in the antioxidant capacity DPPH test (gallic acid or Trolox equivalents) and FRAP test, TPC, and TFC.

In order to correlate these methods, a regression model using the Pearson test was used (Figure 5). The correlation coefficient between TPC and the antioxidant capacity of the ethanolic extracts (i.e., DPPH/TPC) were 0.2430 and −0.0851 for air-dried and freeze-dried, respectively. Similarly, the coefficients for the pair of FRAP/TPC were 0.2887 for air-dried and 0.1082 for freeze-dried. These values indicated no correlation between those parameters. Therefore, the radical-scavenging capacity and ferric-reducing activity of ethanolic extracts of *C. quinoa* could not be predicted on the basis of TPC content. TFC and antioxidant capacity were not correlated either. In this regard, antioxidant properties of single compounds within a mixture could vary, but the same levels of phenolics or flavonoids did not necessarily correspond to the same antioxidant response.

All of these results suggested that (1) there were different trends in antioxidant capacity between FRAP and DPPH for freeze-dried samples, but they were similar among air-dried samples; and (2) despite the loss of phenolic content during the air-dried processes (Figure 1), a significant contribution to ferric-reducing capacity remained in those samples (*R* = 0.7423).

### 3.4. Metabolite Fingerprinting

According to the recorded mass spectra (positive and negative modes) for the main chromatographic peaks in overall extracts, in comparison to those compounds reported for the *Chenopodium* genus, different types of metabolites were annotated, such as saponins, flavonols, coumarins, and sesquiterpenes (Table 1). The identified flavonols have been widely described as antioxidant activity originating in *C. quinoa* [38,57].

**Figure 6:**
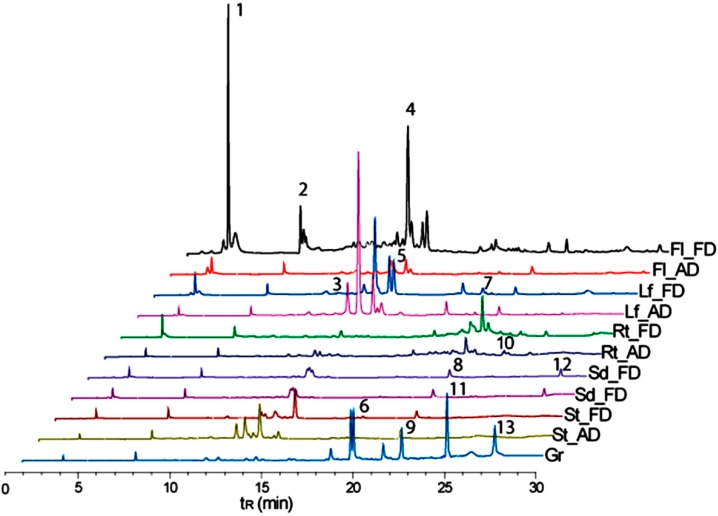
Chromatographic profiles comparing the content of compounds in the extracts from all analyzed plant parts and their respective drying treatment. Methods: AD = air-drying, FD = freeze-drying. Plant parts: Leaves (Lf), stems (St), roots (Rt), seeds (S), flowers (Fl), granules (Gr).

According to the relative abundance (calculated from peak areas of each compound and expressed as %) for each chromatographic profile, the annotated compounds exhibited different distributions depending on the plant part (Figure 6). Thus, major compound diversity was observed in freeze-dried flowers, comprised of coumarin-type compounds (e.g., scopoletin (**1**, 10.2%)), sesquiterpenes (e.g., naphthalenone (**2**, 2.3%)), and flavonols (e.g., rhamnetin 3-glucoside (**4**, 8.5%)). In the case of the leaves, the main compounds were flavonols, which had no significant variance when samples from the different drying methods were compared. Thus, rhamnetin 3-glucoside (**4**) exhibited a relative abundance of 14.0% in freeze-dried samples and 22.2% in air-dried samples. The unidentified compound **5** reached 4.6% in freeze-dried samples and 7.5% in air-dried samples. However, kaempferol 3-*O*-sophoroside (**3**) showed a reduction from 15.6% in freeze-dried samples to 7.5% in air-dried samples. Upon comparing the different culture stages, an increase in glycosylated flavonols was observed, which was related to the rise of the temperature in the seasons. The stems showed the same performance as that of the leaves. Thus, a higher relative abundance of compounds **3**, **4**, and **5** was present in the air-dried samples compared to the freeze-dried samples. In roots, the presence of compound **7** was representative at different quantities (3.6% for freeze-dried and 2.9% for air-dried samples).

The nonpolar fraction obtained from starch granules (after chloroform cold-soaking extraction) revealed the presence of saponin compounds **9** and **11**, reported previously as found in other plant parts (flowers, fruits, seeds, and seed coats). Saponins were detected at low quantities in nonpolar extracts obtained from these organs [41]. In the present work, this kind of compound was detected at significant relative abundances within the chloroform extract, indicating that that constituted an important fraction of the organic-soluble components from the starch granules. These compounds have been widely described for *C. quinoa* seeds [58,59] and studied due to their antifungal potential and hemolytic qualities. However, these compounds are responsible for the bitter taste of *C. quinoa*, but can be removed or reduced by an extrusion and roasting process [60].

#### Multivariate Statistical Analysis

**Figure 7:**
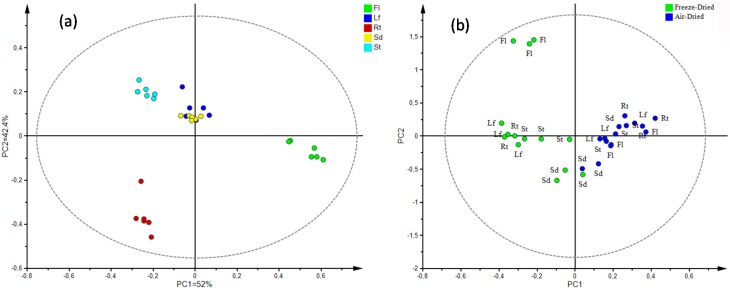
(**a**) Orthogonal partial least squares discriminant analysis (OPLS-DA) derived score plot for individual LC/MS samples tested, supervised by plant parts. Unit variance (UV) autoscaling. (**b**) OPLS-DA score plot for individual LC/MS samples tested, discriminated by drying process method. Internal cross-validation produced *Q*^2^ = 0.892. UV autoscaling.

The multivariate analysis was aimed at determining the differences in the metabolite composition among the distinct plant parts and the influence of the drying treatment (air- and freeze-drying) on the secondary metabolites with antioxidant properties. Under this criterion, a supervised analysis was then performed, taking into account these same parameters for discrimination. The data were mean-centered and scaled to unit variance (UV). This scaling method consists of the most balanced tool in metabolomics-based analysis, since it gives the same priority level to all features (i.e, metabolites), independent of their relative abundance [61]. Hence, in the principal component (PC)1 versus PC2 score plot shown in Figure 7a, the evident differences are distributed along PC1 and PC2, which explained 94.4% of the observed variance. The scores associated with the flowers, forming an independent group, exhibited the best antioxidant activity.

The supervised analysis discriminated by drying method (Figure 7b, air-dried vs freeze-dried, *R*^2^*_cum_* = 0.95), exhibiting a general difference between the groups. Nevertheless, the points corresponding to seeds (at plot center) linked the groups, indicating the presence of the same metabolites in samples processed by the two methods: That is, the compounds in air-dried seeds were kept in the freeze-dried ones.

**Figure 8:**
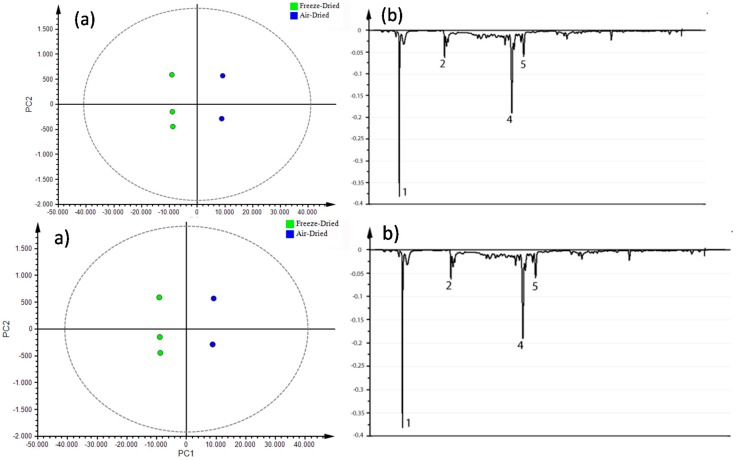
(**a**) OPLS-DA score plot for individual flower extracts profiled by LC/MS used for testing, discriminated by drying process method. Internal cross-validation produced a *Q*^2^ of 0.890, clearly well within acceptable limits. Centered autoscaling. (**b**) Loading line of metabolites detected by LC/MS with annotated compounds: Scopoletin (**1**), naphthalenone (**2**), rhamnetin 3-glucoside (**4**), and unidentified compound (**5**) with an *m/z* of 492.

The extracts exhibiting the best antioxidant activity, TPC, and TFC in the whole dataset were the freeze-dried flowers. Therefore, an additional OPLS-DA model was built using such flower extracts, classified by drying method. The resulting score plot was then obtained (Figure 8a, *R*^2^ = 0.96), indicating a clear separation of flower samples according to drying method. Thus, the corresponding loading line (Figure 8b) led to the identification of the most influential compounds for discrimination between drying methods. Such compounds were found to be a glycosylated flavonoid (rhamentin 3-glucoside), a coumarin (scopoletin), and an aromatic sesquiterpene. This finding indicated that freeze-drying *C. quinoa* flowers favored the extraction of antioxidant compounds in comparison to air-drying.

In seeds (the commercial product of quinoa), the supervised analysis did not generate any defined groups, which indicated that the drying method had no influence on the metabolite profiles of seeds (data not shown). However, there is no loss of phenolic compounds in quinoa seeds when they are exposed to high temperatures by heating, e.g., during cooking [62].

## 4. Conclusions

The TPC and TFC showed a similar trend, since higher values were obtained for freeze-dried samples than for air-dried ones. However, the scatter diagram showed lower values for Pearson correlation coefficients between TPC and TFC with regard to the free radical scavenging (DPPH) but barely higher values for ferric-reducing antioxidant power (FRAP) for both drying methods. From this fact, the observed free radical scavenging activity could not be exclusively associated with phenols and flavonoids. The antioxidant capacity was retained in extracts, independent from the drying process. Considering the reasonable results for separate plant part-derived extracts, the flower extracts showed the highest values for TPC and TFC for both drying processes. Regarding the leaf extracts, they showed the best performance in the FRAP assay for those samples collected at 6 months of growth. Taking into account that this was the stage just before the seeds were harvested, it should be considered that leaves collected together with seeds could be an important ferric-reducing product. In the case of the seeds, they exhibited no changes in the fingerprints upon comparison of the seed samples processed by the two different drying methods. Finally, some glycosylated and galactosylated flavonols were annotated in different plant parts, such as rhamnetin 3-glucoside, kaempferol 3-*O*-sophoroside, and kaempferol 3-rhamnosyl-(1→2)-galactoside. The results showed that flavonoids and phenolics in *C. quinoa* could change during growth between plant parts and drying method, whereas antioxidant capacity exhibited no significant differences in such factors. These changes need to be taken into consideration when comparing the production/accumulation of these compounds in *C. quinoa*. Therefore, this study highlights the basis for further investigations in the course of the selective production of antioxidant compounds from this plant. The appropriate selection of the most suitable crop conditions and postharvest processes with regard to the highest presence of beneficial phytoconstituents is very important for functional food production.

## Figures and Tables

**Table 1:** Peak annotations in *Chenopodium quinoa*-derived extracts after LC/MS analysis.

No ^a^	t_R_^b^ (min)	[M + H]^+^ *m/z*	[M − H]^−^ *m/z*	Annotated Metabolites	Molecular Formula	Exact Mass *m/z*	Type
**1**	3.8		191	scopoletin	C_10_H_8_O_4_	192.0422	C
**2**	7.7	294		naphthalenone	C_17_H_26_O_4_	294.1831	T
**3**	12.9		609	kaempferol 3-*O*-sophoroside	C_27_H_30_O_16_	610.1533	F
**4**	13.5	479	477	rhamnetin 3-glucoside	C_22_H_22_O_12_	478.1111	F
**5**	14.5	493	491	unidentified	-	-	*
**6**	19.5		810	quinoasaponin 3	C_42_H_66_O_15_	810.4401	S
**7**	20.3		647	unidentified	C_27_H_30_O_16_	-	*
**8**	21.2		592	kaempferol 3-rhamnosyl-(1→2)-galactoside	C_27_H_30_O_15_	594.1584	F
**9**	22.2	959		quinoasaponin 2	C_48_H_78_O_19_	958.5137	S
**10**	23.0		815	unidentified	-	-	●
**11**	24.7		792	chikusetsusaponin IVa	C_42_H_66_O_14_	794.4452	S
**12**	27.0		285	kaempferol	C_15_H_10_O_6_	286.0477	●
**13**	27.3	411		unidentified	-	-	*

C: Coumarin, F: Flavonol, S: Saponin, T: Sesquiterpene. * Mass spectra data without a match after database searching (restricted to *Chenopodium* genus). Identified compounds found exclusively in seeds. ^a^ Numbering according to Figure 6. ^b^ Retention time (t_R_).

## References

[1] Alvarez-Jubete L., Wijngaard H., Arendt E.K., Gallagher E. (2010). Polyphenol composition and in vitro antioxidant activity of amaranth, quinoa buckwheat and wheat as affected by sprouting and baking. Food Chem..

[2] Paśko P., Bartoń H., Zagrodzki P., Gorinstein S., Fołta M., Zachwieja Z. (2009). Anthocyanins, total polyphenols and antioxidant activity in amaranth and quinoa seeds and sprouts during their growth. Food Chem..

[3] Paśko P., Sajewicz M., Gorinstein S., Zachwieja Z. (2008). Analysis of selected phenolic acids and flavonoids in *Amaranthus cruentus* and *Chenopodium quinoa* seeds and sprouts by HPLC. Acta Chromatogr..

[4] Bigliardi B., Galati F. (2013). Innovation trends in the food industry: The case of functional foods. Trends Food Sci. Technol..

[5] Blades M. (2000). Functional foods or nutraceuticals. Nutr. Food Sci..

[6] Tur J.A., Bibiloni M.M. (2016). Functional Foods. Encyclopedia of Food and Health.

[7] Nimse S.B., Pal D. (2015). Free radicals, natural antioxidants, and their reaction mechanisms. RSC Adv..

[8] Creus E.G. (2004). Phenolic compounds. An analysis of its health benefits. Offarm Farm. Soc..

[9] Delgado-Olivares L., Betanzos-Cabrera G., Sumaya-Martínez M.T. (2010). Importance of dietary antioxidants in the reduction of oxidative stress. Investig. Cienc..

[10] King A.M.Y., Young G. (1999). Characteristics and occurrence of phenolic phytochemicals. J. Am. Diet. Assoc..

[11] Sreelatha S., Padma P.R. (2009). Antioxidant activity and total phenolic content of *Moringa oleifera* leaves in two stages of maturity. Plant Foods Hum. Nutr..

[12] Mutasim M.K., Hussein M.D.A.N., Khalid M.A.E., Hany A.E.S., Eltayb A. (2011). Dedifferentiation of leaf explants and antileukemia activity of an ethanolic extract of cell cultures of *Moringa oleifera*. Afr. J. Biotechnol..

[13] Gutteridge J.M.C., Halliwell B. (2002). Antioxidant Protection and Ixygen Radical Signaling. Reactive Oxygen Species in Biological Systems.

[14] Mariani E., Polidori M.C., Cherubini A., Mecocci P. (2005). Oxidative stress in brain aging, neurodegenerative and vascular diseases: An overview. J. Chromatogr. B.

[15] Gawlik-Dziki U., Świeca M., Sułkowski M., Dziki D., Baraniak B., Czyż J. (2013). Antioxidant and anticancer activities of *Chenopodium quinoa* leaves extracts: In vitro study. Food Chem. Toxicol..

[16] San-Miguel A., Martin-Gil F.J. (2009). Importance of reactive oxygen species (free radicals) and antioxidants in clinics. Gac. Med. Bilbao.

[17] Chandrasekara A., Shahidi F. (2011). Inhibitory activities of soluble and bound millet seed phenolics on free radicals and reactive oxygen species. J. Agric. Food Chem..

[18] Gebicki J.M. (2016). Oxidative stress, free radicals and protein peroxides. Arch. Biochem. Biophys..

[19] Wojtunik-Kulesza K.A., Oniszczuk A., Oniszczuk T., Waksmundzka-Hajnos M. (2016). The influence of common free radicals and antioxidants on development of Alzheimer’s Disease. Biomed. Pharmacother..

[20] Lobo V., Patil A., Phatak A., Chandra N. (2010). Free radicals, antioxidants and functional foods: Impact on human health. Pharmacogn. Rev..

[21] Kaur C., Kapoor H.C. (2001). Antioxidants in fruits and vegetables—The millennium’s health. Int. J. Food Sci. Technol..

[22] Shahidi F., Shahidi F. (2015). Antioxidants: Principles and applications. Woodhead Publishing Series in Food Science, Technology and Nutrition.

[23] Pandey K.B., Rizvi S.I. (2009). Plant polyphenols as dietary antioxidants in human health and disease. Oxid. Med. Cell. Longev..

[24] Berti C., Ballabio C., Restani P., Porrini M., Bonomi F., Iametti S. (2004). immunochemical and molecular properties of proteins in *Chenopodium quinoa*. Cereal Chem. J..

[25] Mahoney A.W., Lopez J.G., Hendricks D.G. (1975). Evaluation of the protein quality of quinoa. J. Agric. Food Chem..

[26] Repo-Carrasco R., Espinoza C., Jacobsen S.-E. (2003). Nutritional value and use of the andean crops quinoa (*Chenopodium quinoa*) and kañiwa (*Chenopodium pallidicaule*). Food Rev. Int..

[27] Woldemichael G.M., Wink M. (2001). Identification and Biological activities of triterpenoid saponins from *Chenopodium quinoa*. J. Agric. Food Chem..

[28] Jacobsen S.E., Mujica A., Ortiz R. (2003). The importance of Andean crops. Fermentum.

[29] Mujica A., Jacobsen S.-E. (2007). Quinoa (*Chenopodium quinoa* Willd.) and its wild relatives. Botánica Económica los Andes Cent..

[30] Romo S., Rosero A., Forero C., Céron E. (2006). Nutritional potential of quinoa flours (*Chenopodium quinoa* w) piartal variety in the Colombian Andes Part One. Biotecnol. en el Sect. Agropecu. y Agroindustrial.

[31] Brito I.L., de Souza E.L., Felex S.S.S., Madruga M.S., Yamashita F., Magnani M. (2015). Nutritional and sensory characteristics of gluten-free quinoa (*Chenopodium quinoa* Willd)-based cookies development using an experimental mixture design. J. Food Sci. Technol..

[32] Lutz M., Martínez A., Martínez E.A. (2013). Daidzein and Genistein contents in seeds of quinoa (*Chenopodium quinoa* Willd.) from local ecotypes grown in arid Chile. Ind. Crops Prod..

[33] Alvarez-Jubete L., Arendt E.K., Gallagher E. (2009). Nutritive value and chemical composition of pseudocereals as gluten-free ingredients. Int. J. Food Sci. Nutr..

[34] Alvarez-Jubete L., Arendt E.K., Gallagher E. (2010). Nutritive value of pseudocereals and their increasing use as functional gluten-free ingredients. Trends Food Sci. Technol..

[35] Nowak V., Du J., Charrondière U.R. (2016). Assessment of the nutritional composition of quinoa (*Chenopodium quinoa* Willd.). Food Chem..

[36] Bai J.C., Fried M., Corazza G.R., Schuppan D., Farthing M., Catassi C., Greco L., Cohen H., Ciacci C., Eliakim R. (2013). World Gastroenterology Organisation Global Guidelines on Celiac Disease. J. Clin. Gastroenterol..

[37] Fischer S., Wilckens R., Jara J., Aranda M. (2013). Variation in antioxidant capacity of quinoa (*Chenopodium quinoa* Will) subjected to drought stress. Ind. Crops Prod..

[38] Hirose Y., Fujita T., Ishii T., Ueno N. (2010). Antioxidative properties and flavonoid composition of *Chenopodium quinoa* seeds cultivated in Japan. Food Chem..

[39] Tang Y., Li X., Zhang B., Chen P.X., Liu R., Tsao R. (2015). Characterisation of phenolics, betanins and antioxidant activities in seeds of three *Chenopodium quinoa* Willd. genotypes. Food Chem..

[40] Kuljanabhagavad T., Wink M. (2009). Biological activities and chemistry of saponins from *Chenopodium quinoa* Willd. Phytochem. Rev..

[41] Kuljanabhagavad T., Thongphasuk P., Chamulitrat W., Wink M. (2008). Triterpene saponins from *Chenopodium quinoa* Willd. Phytochemistry.

[42] Bernal F.A., Cuca-Suárez L.E., Yamaguchi L.F., Coy-Barrera E. (2013). LC-DAD-UV and LC-ESI-MS-based analyses, antioxidant capacity, and antimicrobial activity of a polar fraction from *Iryanthera ulei* leaves. Rec. Nat. Prod..

[43] Mad T., Sterk H., Mittelbach M., Rechberger G.N. (2006). Tandem mass spectrometric analysis of a complex triterpene saponin mixture of *Chenopodium quinoa*. J. Am. Soc. Mass Spectrom..

[44] Zhang D., Hamauzu Y. (2004). Phenolics, ascorbic acid, carotenoids and antioxidant activity of broccoli and their changes during conventional and microwave cooking. Food Chem..

[45] Ismail A., Marjan Z., Foong C. (2004). Total antioxidant activity and phenolic content in selected vegetables. Food Chem..

[46] Toor R.K., Savage G.P. (2006). Effect of semi-drying on the antioxidant components of tomatoes. Food Chem..

[47] Roy M.K., Takenaka M., Isobe S., Tsushida T. (2007). Antioxidant potential, anti-proliferative activities, and phenolic content in water-soluble fractions of some commonly consumed vegetables: Effects of thermal treatment. Food Chem..

[48] Miranda M., Vega-Gálvez A., López J., Parada G., Sanders M., Aranda M., Uribe E., Scala K. (2010). Di Impact of air-drying temperature on nutritional properties, total phenolic content and antioxidant capacity of quinoa seeds (*Chenopodium quinoa* Willd.). Ind. Crops Prod..

[49] Martín-Cabrejas M.A., Aguilera Y., Pedrosa M.M., Cuadrado C., Hernández T., Díaz S., Esteban R.M. (2009). The impact of dehydration process on antinutrients and protein digestibility of some legume flours. Food Chem..

[50] Qu W., Pan Z., Ma H. (2010). Extraction modeling and activities of antioxidants from pomegranate marc. J. Food Eng..

[51] Repo-Carrasco-Valencia R., Hellström J.K., Pihlava J.-M., Mattila P.H. (2010). Flavonoids and other phenolic compounds in Andean indigenous grains: Quinoa (*Chenopodium quinoa*), kañiwa (*Chenopodium pallidicaule*) and kiwicha (*Amaranthus caudatus*). Food Chem..

[52] Asao M., Watanabe K. (2010). Functional and bioactive properties of quinoa and amaranth. Food Sci. Technol. Res..

[53] Weinig C., Gravuer K.A., Kane N.C., Schmitt J. (2004). Testing adaptive plasticity to UV: Costs and benefits of stem elongation and light-induced phenolics. Evolution (N. Y.).

[54] Vollmannová A., Margitanová E., Tóth T., Timoracká M., Urminská D., Bojňanská T., Čičová I. (2013). Cultivar influence on total polyphenol and rutin contents and total antioxidant capacity in buckwheat, amaranth, and quinoa seeds. Czech J. Food Sci..

[55] Repo-Carrasco R., Encina R. (2008). Determination of the antioxidant capacity and phenolic compounds of Andean cereals: Quinoa (*Chenopodium quinoa*), kañiwa (*Chenopodium pallidicaule*) and kiwicha (*Amaranthus caudatus*). Rev. Soc. Quim. Perú.

[56] Nsimba R.Y., Kikuzaki H., Konishi Y. (2008). Antioxidant activity of various extracts and fractions of *Chenopodium quinoa* and *Amaranthus* spp. seeds. Food Chem..

[57] Zhu N., Sheng S., Li D., Lavoie E.J., Karwe M.V., Rosen R.T., Ho C.T. (2001). Antioxidative flavonoid glycosides from quinoa seeds (*Chenopodium quinoa* Willd). J. Food Lipids.

[58] Lindeboom N., Chang P.R., Tyler R.T. (2004). Analytical, biochemical and physicochemical aspects of starch granule size, with emphasis on small granule starches: A review. Starch.

[59] Lorenz K. (1990). Quinoa (Chenopodium quinoa) starch: Physico-chemical properties and functional characteristics. Starch.

[60] Brady K., Ho C.-T., Rosen R.T., Sang S., Karwe M. (2007). V Effects of processing on the nutraceutical profile of quinoa. Food Chem..

[61] Palama T.L., Khatib A., Choi Y.H., Côme B., Fock I., Verpoorte R., Kodja H. (2011). Metabolic characterization of green pods from *Vanilla planifolia* accessions grown in La Réunion. Environ. Exp. Bot..

[62] Chlopicka J., Pasko P., Gorinstein S., Jedryas A., Zagrodzki P. (2012). Total phenolic and total flavonoid content, antioxidant activity and sensory evaluation of pseudocereal breads. LWT-Food Sci. Technol..

